# Composition, Microbiota, Mechanisms, and Anti-Obesity Properties of Rice Bran

**DOI:** 10.3390/foods12061300

**Published:** 2023-03-18

**Authors:** Bhagavathi Sundaram Sivamaruthi, Karthikeyan Alagarsamy, Subramanian Thangaleela, Muruganantham Bharathi, Periyanaina Kesika, Chaiyavat Chaiyasut

**Affiliations:** 1 Office of Research Administration, Chiang Mai University, Chiang Mai 50200, Thailand; sivamaruthi.b@cmu.ac.th; 2 Innovation Center for Holistic Health, Nutraceuticals, and Cosmeceuticals, Faculty of Pharmacy, Chiang Mai University, Chiang Mai 50200, Thailand; 3 Department of Microbiology (Aided), PSG College of Arts and Science, Avinashi Road, Civil Aerodrome Post, Coimbatore 641014, Tamil Nadu, India

**Keywords:** rice bran, rice bran oil, microbiome, obesity, γ-oryzanol, tocopherols, tocotrienols

## Abstract

Rice is a major cereal crop and a staple food for nearly 50% of people worldwide. Rice bran (RB) is a nutrient-rich by-product of rice processing. RB is rich in carbohydrates, fibers, proteins, lipids, minerals, and several trace elements (phosphorus, calcium, magnesium, potassium, and manganese). The extraction process and storage have influenced RB extracts and RB oil’s quality. The RB composition has also varied on the rice cultivars. The color of RB indicates the richness of the bioactive compounds, especially anthocyanins. γ-oryzanol, tocopherols, tocotrienols, and unsaturated fatty acids are major components of RB oil. It has been established that RB supplementation could improve the host’s health status. Several preclinical and clinical studies have reported that RB has antioxidant, anticancer, anti-inflammatory, anticolitis, and antidiabetic properties. The beneficial biological properties of RB are partially attributed to its ability to alter the host microbiome and help to maintain and restore eubiosis. Non-communicable diseases (NCDs), including heart disease, diabetes, cancer, and lung disease, account for 74% of deaths worldwide. Obesity is a global health problem and is a major reason for the development of NCDs. The medical procedures for managing obesity are expensive and long-term health supplements are required to maintain a healthy weight. Thus, cost-effective natural adjuvant therapeutic strategy is crucial to treat and manage obesity. Several studies have revealed that RB could be a complementary pharmacological candidate to treat obesity. A comprehensive document with basic information and recent scientific results on the anti-obesity activity of RB and RB compounds is obligatory. Thus, the current manuscript was prepared to summarize the composition of RB and the influence of RB on the host microbiome, possible mechanisms, and preclinical and clinical studies on the anti-obesity properties of RB. This study suggested that the consumption of RB oil and dietary RB extracts might assist in managing obesity-associated health consequences. Further, extended clinical studies in several ethnic groups are required to develop dietary RB-based functional and nutritional supplements, which could serve as an adjuvant therapeutic strategy to treat obesity.

## 1. Introduction

*Oryza sativa* (rice) is a common food crop worldwide and is cultivated in several countries. Rice is one of the major sources of carbohydrates, especially in low-income countries [1]. Rice bran (RB) is the by-product of the rice milling process and a major food waste. RB contains fat (12 to 25%), starch (10 to 20%), protein (10 to 16%), celluloses (10 to 12%), hemicellulose (8 to 11%), fiber (6 to 15%), reducing sugar (3 to 8%), ash (6.5 to 10%), and phenolic acids, γ-oryzanol, and tocopherols [2,3]. RB has minerals such as magnesium, iron, and phosphorus [4]. Full fat RB contains 18–22% oil and bioactive phytochemicals such as oryzanols, phytosterols, tocotrienols, squalene, polycosanols, and phytic acid, ferulic acid, and inositol hexaphosphate [5]. Thus, RB could be deemed a sustainable, functional bioactive candidate for several applications, including the pharmacology and cosmetic industries. RB could reduce the risk of several chronic diseases [6].

RB is mainly used for oil extractions, and the processing of RB yields several by-products, including RB fatty acid distillate, wax, and defatted RB. RB oil (RBO) composition varies depending on the extraction methods [7]. RBO contains nutritionally beneficial compounds like sterols, tocopherols, tocotrienols, γ-oryzanol, range of fats and other bioactive compounds [8]. γ-oryzanol has a beneficial effect on lipid metabolism [9], glucose metabolism [10], and the cardiovascular system [11]. RB also contains gamma-aminobutyric acid (GABA), a known neurotransmitter [8].

Obesity is a global health problem and is a chronic disease characterized by the excessive accumulation of body fat. Exercise, diets (carbohydrate-restricted diets, carbohydrate-reduced high-protein diets, low-fat diets, fiber-rich Mediterranean diets), bariatric surgery, medicines, and micro-RNA-based treatments are clinical strategies to prevent and treat obesity [12]. Besides the above-mentioned strategies, maintaining healthy gut microbiota and psychological management also influence the obese condition [12]. Phenolic compounds also play an important role in regulating obesity and gut microbiota [13]. Gut microbiota influences almost all of the human physiological, biological, and psychological conditions. Gut microbiota is a crucial factor in intestinal barrier function and homeostasis, regulation of metabolism, and the immune system. Gut dysbiosis is associated with cardiovascular disease, diabetes, cancers, inflammatory diseases, and obesity [14]. Diet influences gut microbial composition [15], so bioactive supplements in the diet may positively regulate microbiota and body weight [16]. The phenolic compounds could regulate the metabolic syndrome through gut microbiota [17]. Studies have shown that the dietary supplementation of RB altered Enterobacteriaceae, Streptococcaceae, Enterococcaceae, Lachnospiraceae, and Ruminococcaceae [18], *Bacteroidetes, Coprococcus, Lachnobacterium, Firmicutes*, *Ruminococcus*, and *Ethanoligenens* [19], and improved gut microbial diversity [20].

Clinical studies have shown that dietary supplementation of RB (regular diet + RB extract) could improve lipid profile, blood pressure, and serum glucose level, and the inflammatory system of obese subjects [21,22]. The intervention of RB, an energy/carbohydrate diet, and plant sterols may improve the obesity-associated parameters in obese people [23,24].

The objective of the current study was to summarize the composition of RB, the influences of RB supplementation on gut microbiota, the anti-obesity properties of RB, and the mechanisms of RB-mediated anti-obesity properties.

## 2. Phytochemical Composition of Rice Bran

Rice is rich in carbohydrates, proteins, fatty acids, vitamins, traces of minerals, phenolic acids, terpenoids, steroids, and alkaloids [25,26,27]. The phytochemicals of various rice types are categorized into carotenoids, phenolics, alkaloids, nitrogen, and organosulfur-containing compounds. The phenolic components are sub-divided into phenolic acids, flavonoids, tannins, and coumarins [28]. RB contains a variety of phytochemicals that benefit human health [29]. The composition and percentage of RB differ according to the rice cultivars, pre-treatment for milling, milling methods, degree of milling, and extraction methods [30]. The milling process of rice yields RB and various by-products that contain vitamin E, thiamine, niacin, and minerals such as calcium, aluminum, chlorine, iron, magnesium, manganese, sodium, potassium, phosphorus, and zinc [31,32]. Among the by-products, RB possesses non-saponifiable lipids, including γ-oryzanol, vitamin E, polyphenols such as ferulic acid, caffeic acid, salicylic acid, and phytosterols β-sitosterol [33], with high nutritive value and many health benefits [34]. These secondary metabolites of RB have various physiological and ecological functions, such as antimicrobial, insecticidal, and allelopathic activities, and have cytotoxic, antioxidant, antidiabetic, antitumor, hypocholesterolemic, and neuroprotective properties [35,36]. Most phytocompounds are available in the lipid fraction and are called RB oil (RBO) [37].

RBO is a rich source of tocopherols and tocotrienols, widely used in the food, pharmaceutical, and cosmetic industries [38,39]. RBO is highly rich in tocopherols, tocotrienols, and γ-oryzanol. The quality and bioactivities of RBO vary according to the extraction and refining methods. The influence of various extraction methods on total tocols, γ-oryzanol contents, and antioxidant characteristics of Chiang Mai black rice, Mali red rice, and Suphanburi-1 brown rice were reported. The phytochemical composition of RBO extracted by hexane, hot and cold pressed, and supercritical fluid extraction methods were studied. Among these extraction methods, a high quantity of RBO was obtained from the hexane and supercritical fluid extraction methods. The RBO obtained from all three extraction types holds a significantly higher amount of γ tocotrienol. However, the superior RBO quality was sustained, and the phytochemical contents and antioxidant properties remained intact in hexane-extracted samples compared to other methods [7]. It is likely that the RB extract of Chiang Mai black rice, Mali red rice, and Suphanburi-1 brown rice varieties are rich in phenolic acids, flavonoids, and anthocyanins [28].

The defatted RB is a rich source of protein (11 to 15%) and is suitable for food applications. The protein content of RB is two-fold higher than other parts of rice. The outer layer of RB has especially high protein content, and it will reduce with the increasing milling process. The protein concentration differs among the rice varieties due to different mechanisms of protein accumulation in the grains [40]. RB protein is nutritionally superior with a prominent amino acid profile and is rich in lysine, aspartic acid, glycine, arginine, alanine, cystine, histidine, and threonine compared to other cereal proteins [41]. RB proteins are mostly the storage protein and including the albumin, globulin, glutelin, and prolamin with high functional properties, helps to store nitrogen, carbon, and sulfur for grain and results in dense deposits called protein bodies [42]. RB proteins are used in the food industry as a food ingredient and additional food formulations, and as a natural emulsifier in food products, because of their nutritional quality [43,44].

RB contains approximately 12% dietary fiber, mostly insoluble cellulose and hemicellulose [4,45], and traces of soluble fibers such as pectin and β-glucan [46]. Various varieties of white RB contain phenolic compounds, including ferulic acid, isoferulic acid, vanillic acid, *p*-coumaric acid, sinapic and syringic acids, and flavonoids such as rutin, myricetin, and quercetin-3-glucuronide [47,48]. Some studies revealed the presence of tocols in white RB, which includes α-tocopherols, α-tocotrienols, γ-tocopherols, γ-tocotrienols, δ-tocopherols, δ-tocotrienols, and γ-oryzanols, and squalene and phytosterols such as stigmasterol, campesterol, and β-sitosterol [49,50]. γ-oryzanol is present in the form of steryl ferulate, and the fraction of γ-oryzanol in RBO varies depending on the rice cultivar and extraction methods [51]. The RB metabolome study identified 453 phytochemicals, with 46% of them amino acids, cofactors, vitamins, and secondary metabolites with health benefits. The metabolites were classified as anti-inflammatory (*n* = 35), antimicrobial (*n* = 15), antihypertensive (*n* = 12), anticancer (*n* = 11), antihyperlipidemic (*n* = 8), antihyperglycemic (*n* = 6), and 2 anti-obesogenic (*n* = 2) compounds [29]. The saturated fatty acids in RB act as antioxidants and anticancer compounds [52]. Oryzanol possesses very high antioxidant properties that tocopherols lack. γ-oryzanol shows a structural similarity with cholesterol and helps reduce oxidative stress and maintain cell functionality [53]. Germinated RB is rich in γ-amino butyric acid (GABA), dietary fiber, ferulic acid, tocotrienols, and γ-oryzanol [54]. RB is also a high-quality protein with more digestibility. It is hypoallergenic [55], including albumin, globulin, glutelin, and prolamin, and its RB protein fractions differ according to rice varieties [42].

The phenolic acids in rice are classified into soluble-free, soluble-conjugated, and insoluble-bound forms. The insoluble bound forms of phenolic acid are covalently attached to the structural components of cells, which are cellulose, hemicellulose, lignin, pectin, and other proteins [56]. Generally, pigmented rice contains more phenolic acids than non-pigmented rice varieties [57]. Rice varieties of *Oryza sativa*, *O. japonica*, and *O. sativa* sp. *indica* from different regions of China were evaluated for their phenolic acids content. The results showed that 12 phenolic compounds are present in all rice varieties. *O. japonica* has a higher phenolic content than other studied varieties [58]. The free, bound, and total phenolic and flavonoid compounds differ in chemical composition and antioxidant activities in defatted RB and their soluble and insoluble fibers were evaluated by Zhao et al. The soluble fiber from defatted RB has low total phenolic and total flavonoid contents. The insoluble fiber from defatted RB has low free phenolics and high bound phenolics. They found 17 monomeric phenolic compounds in defatted RB, including gallic acid, syringic acid, vanillin, epicatechin, *p*-coumaric acid, ferulic acid, sinapic acid, quercitrin, isoquercitrin, caffeic acid methyl, and ferulic acid methyl [59].

Sompong et al. reported the phytochemical content of nine red and three black rice cultivars from Thailand, China, and Sri Lanka. They identified cyanidin-3-glucoside and peonidin-3-glucoside as predominant anthocyanins in black rice. The highest total phenolic content was observed in the red Thai rice variety (Bahng Gawk). Moreover, red rice varieties showed the major free form of ferulic acid, protocatechuic acid, and vanillic acid. In contrast, black rice varieties were rich in protocatechuic acid, then ferulic and vanillic acid [60]. The Hashemi RB extracts are abundant with phenolic contents, including ferulic, gallic, and chlorogenic acids [27]. Thai rice cultivars contain phenolic acids such as caffeic acid, chlorogenic acid, protocatechuic acid, *p*-hydroxybenzoic acid, and syringic acid, and *p*-coumaric acid, anthocyanins, tocols and γ-oryzanol. The concentration of phenolic acids differs among rice varieties [61]. The impact of extraction methods on the yield of anthocyanins was studied in nine different Thai rice cultivars, including Hawm nil, Hawm kanya, and Kum (black grains), Sang yod and Red jasmine (red grains), and Hawm ubon, Lao tek, Jasmine rice 105, and Sin lek (white grains). The results showed that the extraction methods affected the phytochemical content of the extracts. Insoluble phenolic compounds were significantly higher than soluble phenolic compounds. Among these rice varieties, black grains showed higher anthocyanins and phenolic compounds than red and white grains [62]. The purple rice bran has a high proportion of anthocyanins [40].

Huang and Lai investigated the profiles of free and bound phenolics and flavonoid compounds in six pigmented rices’ outer and inner RB. The 80% ethanol extracts of red rice varieties from Taibalang, Taiwan, and Thailand were reported to have proanthocyanin, anthocyanin, vitamin E, and γ-oryzanol. Proanthocyanins were found in red RB and were absent in black RB. HPLC with photodiode array/electrospray ionization tandem mass spectrometry identified protocatechualdehyde in the bound fraction of red RB. The crude lipid, protein, ash, and total dietary fiber were higher in outer RB than inner RB. Moreover, phenolics and flavonoids in free fractions were higher than inbound fractions. The colored RB contains α, β, γ, and δ- tocopherol and α, γ, and δ tocotrienol [63].

Flavonoids are another group of secondary metabolites present in rice and are classified as flavones, flavanols, flavanones, flavanonols, and anthocyanins with extraordinary antioxidant capacities [64]. Flavones from the Njavara rice variety had a potential cytotoxicity effect against cancer cells [65]. Sakuranetin is a flavanone type of phytoalexin that works against plant pathogens. Moreover, it acts as a pharmaceutical agent that induces adipogenesis in 3T3-L1 cells, thus regulating glucose homeostasis in animals [66], and has anti-inflammatory [67], antimutagenic [68], antileishmanial, and antitrypanosomal activities [69]. The differences in flavonoid contents in different white, red, and black-pigmented rice varieties were reported. Black RB has a high content of cyanidin-3-glucoside, peonidin-3-glucoside, quercetin, dihydromyricetin, naringin, and taxifolin. Red RB is rich in catechin and epicatechin. The red and black RB extracts exhibited higher antioxidant activity than the white RB extract [70].

Anthocyanins are water-soluble pigments belonging to flavonoids responsible for colors in plant tissues with significant antioxidant activities and rich in pigmented rice varieties. Cyanidin, cyanidin-3-O-gentiobioside, cyanidin-3-O-glucoside, cyanidin-3-O-rutinoside, peonidin, and peonidin-3-O-glucoside are certain types of anthocyanins identified in black rice kernels [64,71]. The black rice *O. sativa* L. indica is rich in anthocyanins, which impart heavy pigmentation to the outer layer of the rice. Black rice contains 95% of total anthocyanins, including delphinidin, pelargonidin, peonidin, cyanidin, malvidin, and petunidin [72,73]. Black rice’s outer bran is richer in anthocyanin than its inner RB. Cyanidin-3-glucoside, peonidin-3-glucoside, and cyanidin-3-rutinoside were found in black and red RB. Black rice contained more protocatechuic and vanillic acid than red RB [63]. Three groups of Thai rice—black glutinous, black non-glutinous, and white non-glutinous rice—were studied for terpenoids. Among these rice varieties, black non-glutinous rice has high monoterpenoids. Monoterpenoid odorants such as limonene, trans-β-ocimene, β-cymene, and linalool are found more in the bran of Thai white jasmine rice Khao Dawk Mali 105 variety. Monoterpenoids (*n* = 19) and sesquiterpenoids (*n* = 9) were found in all three rice varieties. Black non-glutinous RB contains more monoterpenoids and sesquiterpenoids than other RB. The terpenoids (limonene, trans-β-ocimene, β-cymene, linalool, and myrcene) were found in all three RB types. More specifically, trans-β-ocimene and β-cymene were high in white jasmine RB. Myrcene was only detected in black rice varieties [74].

**Figure 1:**
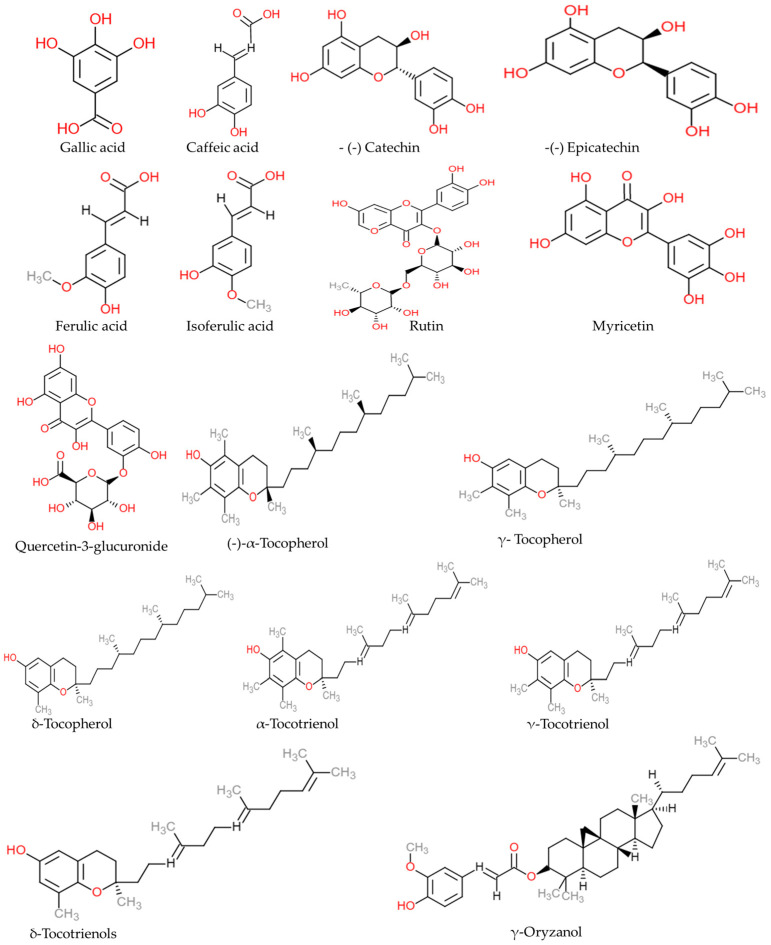
The representative major phytochemicals present in rice bran. Chemical structures were drawn using free online ChemDoodle Web software (https://web.chemdoodle.com/demos/2dsketcher, accessed on 28 February 2023).

The phytochemical composition of RB is influenced by the types of rice cultivars, extraction methods, and milling process. The phytochemical content, extraction, and analysis methods of different rice cultivars are detailed in Table 1. The three-dimensional structure of representative phytochemicals of RB is illustrated in Figure 1.

## 3. Anti-Obesity Properties of Rice Bran

### 3.1. Results of In Vitro Studies

Ethanolic extract of glutinous black RB (EEGBRB), rich in phenolics, flavonoids, and anthocyanins, was studied for its lipolytic property using the 3T3-L1 pre-adipocyte model. EEGBRB treatment reduced lipid accumulation and TG levels in 3T3-L1. EEGBRB treatment also induced lipolysis in 3T3-L1. The study showed that EEGBRB could be a therapeutic agent for managing obesity-associated complications [81]. The hydrolyzed de-oiled RB (DORB) was reported for its anti-obesity property. In detail, DORB was hydrolyzed using Alcalase^®^, and the fractions were further categorized based on their molecular weight. A < 0.65 kDa fraction exhibited the highest level of lipase inhibitory activity. Mass spectrometry analysis revealed that the fraction has the amino acid composition of FYLGYCDY. Further, molecular docking confirmed that the peptide competitively binds with the porcine pancreatic lipase complex. The results indicated that DORB fractions have potent anti-obesity peptides [82]. The defatted red RB was extracted using different concentrations of ethanol. High total flavonoids were shown in 75 and 95% ethanolic extract of defatted red RB. High phenolic content was observed in 50 and 75% ethanolic extract, and these extracts exhibited the highest lipase inhibitory activity in vitro compared to other extracts [83].

To the best of our knowledge, there are no more in vitro studies on the anti-obesity properties of rice bran. Some studies reported the anti-obesity property of rice extract. For example, different solvent extracts of brown rice were screened for pancreatic lipase-inhibitory activity. The results indicated that hexane extract showed the highest inhibition (13.58 ± 0.86%) at 200 g/mL concentration. The inhibitory effects were affected by the concentration of the extracts in vitro [84]. Recently, Barathikannan et al. (2023) demonstrated the anti-obesity activity of *Pediococcus acidilactici* MNL5-mediated fermented brown rice. In vitro pancreatic lipase-inhibitory activity of fermented RB was higher (85.5 ± 1.25%) than raw RB (54.4 ± 0.86%). Moreover, the fermentation process improved the antioxidant capacity of RB. The study also reported that fermented brown rice increased the lifespan and reduced lipid accumulation in *Caenorhabditis elegans* [85].

### 3.2. Results of In Vivo Studies

Several in vivo studies evaluated the anti-obese effects of RB and RB components. Most studies indicated that RB and RB components could ameliorate the obesity and associated co-morbidities. Some of the studies were detailed as follows.

The male Wister rats fed diacylglycerol (DAG)-enriched RB oil and sunflower oil improved the CVD-associated biomarkers. In detail, the supplementation of 20 or 40% of DAG-enriched oils (10% in the diet) for 12 weeks decreased the triacylglycerol (TG), total cholesterol (TC), body fat (BF), C-reactive protein (C-RP), tumor necrosis factor-alpha (TNF-α), and platelet aggregation. Additionally, inducible nitric oxide synthase (iNOS) and cyclooxygenase-2 (COX-2) expression were decreased, and 40%DAG-RB supplementation increased fecal cholesterol excretion. The results indicate that regular intake of 40%DAG-RB could reduce the risk of CVD and improve the serum lipid profile [86].

Obese Zucker rats were supplemented with 1 or 5% RB enzymatic extract (RBEE) for 20 weeks. RBEE supplementation significantly reduced the expression of interleukin (IL)-6, IL-1β, iNOS, and TNF-α in visceral abdominal adipose tissue, while increasing the expression of IL-6 and iNOS in visceral epididymal adipose tissue. RBEE supplementation affected the distribution of adipocytes and effectively reduced adipocyte size [87]. RBEE supplementation also reduced vascular hyperactivity, inflammation, and eNOS (endothelial NOS). Moreover, a significant reduction of superoxide production and down-regulation of NADPH oxidase subunits were also observed in the RBEE-supplemented rats [88]. RBEE-supplementation restored the microvascular function and reduced microvascular inflammation [89]. The studies showed that RBEE could ameliorate obesity-associated proinflammatory responses and vascular issues and restore the function of arteries [88,89,90]. Similarly, supplementation of RBEE improved the lipid profile, adipose tissue, and expression of macrophage polarization in high-fat-diet-induced mice [90].

Male albino rats were supplemented with diacylglycerol-rich bran oil (DAG-RBO) in their regular diet for 28 days. The DAG-RBO-supplemented group showed a reduction in plasma TC and non-HDL-C (non-high density lipid cholesterol) and a reduction in TC, TG, and phospholipids in the mesentery and liver. TG and TL (total lipid) levels were also reduced in the erythrocyte membrane and mesentery. A significant reduction in the HMG-CoA (3-hydroxy-3-methylglutaryl coenzyme A): mevalonate ratio in the liver was observed in the experimental rats. The study suggested that the supplementation of DAG-RBO improved the lipid profile and reduced cholesterol biosynthesis in rats, indicating that DAG-RBO could control the obesity-associated consequences [91].

Triterpene alcohols, β-sitosterol, and campesterol decreased the diet-induced secretion of the glucose-dependent insulinotropic polypeptide (GIP) in C57BL/6J mice. The supplementation of triterpene alcohol and sterol (derived from RB) (TAS) for 23 weeks in mice showed less weight gain than the control. Fat utilization and fatty acid oxidation-related gene expression were higher in TAS-fed mice. The fatty acid synthesis-related gene expression was suppressed in the liver of TAS-fed mice. Cycloartenol and 24-methylene cycloartenol treatment reduced the sterol regulatory element-binding protein (SREBP)-1c expression in HepG2 cells. The results indicated that TAS supplementation could prevent diet-induced obesity in vivo [92].

The supplementation of γ-Oryzanol (OZ) and ferulic acid (FA) derived from RB improved the metabolic syndrome-related parameters in high-fat-diet-induced obese Sprague–Dawley rats. In detail, supplementation of 0.05% FA or 0.16% OZ for 13 weeks improved the obesity, insulin resistance, and lipid profile. In particular, OZ supplementation significantly reduced the hepatic TG level, serum C-reactive protein, and Il-6 and increased the adiponectin level. OZ treatment reduced the intracellular accumulation of TG and expression of stearoyl coenzyme-A desaturase-1. The results suggested that OZ exhibited better protective activity against metabolic syndrome than FA [93].

RB unsaponifiable matter (USM) contains tocopherols and tocotrienols (16.35 mg/g), policosanols (55.14 mg/g), phytosterols (364.25 mg/g), and oryzanols (29.68 mg/g). C57BL/6J mice were supplemented with a high-fat diet and USM (10 or 20, or 50 mg/kg body weight/day) for 6 weeks. USM supplementation effectively decreased the body weight gain, food efficiency ratio, and size of the epididymal fat tissue compared to the control. USM supplementation also reduced the serum TG, TC, LDL-C (low-density lipoprotein cholesterol) levels, cardiac risk factor, and atherogenic index compared to the control. The results showed that USM has antilipidemic activity, which might prevent obesity and cardiovascular disease [94].

Administration of RB water extract (RBWE; 2205 mg/kg/day for 4 weeks) protects the rats from high-fat-diet-induced metabolic changes. In detail, RBWE treatment reduced body weight gain, visceral fat tissue weights, TC, and glucose and malondialdehyde levels compared to the control. Additionally, RBWE treatment increased the expression of eNOS and reduced the expression of NF-kB p65 and CD36 without causing histological changes in the aorta in experimental rats. The results indicated that RBWE has vasoprotective effects in the high-fat-diet-induced obese condition in rats [95]. Similarly, high-fat-diet-fed male Sprague–Dawley rats were supplemented with RBWE (2.205 or 4.410 g/kg/day) for 4 weeks. The changes in diet-induced obesity, hyperglycemia, and glucose tolerances were assessed. RBWE supplementation, in both doses, significantly reduced the body weight and weight gain and controlled the fasting blood glucose and insulin levels compared to high-fat-diet-fed rats. The increased expression of insulin receptor substrate-2 (IRS-2), glucose transporter-2 (GLUT-2), and glucokinase (GK), and decreased expression of sterol regulatory element-binding protein-1c (SREBP-1c), were observed in the pancreas of the RBWE-supplemented rats. RBWE supplementation prevents the formation of fat droplets in acinar cells. The results suggested that RBWE could reduce weight gain, prevent fat accumulation, and improve insulin sensitivity in the high-fat-diet-fed rat model [96].

Red RB extract (RRBE; rich in phenolics, flavonoids, anthocyanins, and proanthocyanidins) supplementation (0.5 or 1 g/kg of RRBE for 6 weeks) protected the mice from the high-fat-diet-induced obese condition. In detail, RRBE supplementation significantly diminishes diet-induced adipocyte hypertrophy and controls lipid accumulation and inflammation, while the body and adipose tissue weights were unchanged. RRBE supplementation also suppressed the expressions of CCAAT/enhancer binding protein-alpha, sterol regulatory element-binding protein-1c, hormone-sensitive lipase, macrophage marker F4/80, NF-kB p65, monocyte chemoattractant protein-1, TNF-α, and iNOS in adipose tissue of the experimental animal. A reduction in serum level of TNF-α was also observed. The results showed that RRBE might improve obesity-associated adipose tissue dysfunction [97]. Similarly, RRBE supplementation improved the serum insulin level, glucose level, and expression of insulin-degrading enzyme (IDE), insulin receptor substrate (IRS), and glucose transporter (GLUT) in high-fat-diet-induced obese mice. RRBE protects the host from diet-induced damage by improving glucose–insulin homeostasis [98].

High-fat/high-cholesterol-diet-fed (HFCD) mice were supplemented with *Weissella koreensis* DB1-mediated fermented RB (HFCD-FRB) or RB (HFCD-RB) (5% FRB or RB) for 10 weeks. FRB supplementation significantly reduced body weight, serum level TG, TC, non-HDL-C, insulin, glucose and leptin, TG in the liver and adipose, and liver and white fat masses. Serum-level adiponectin and HDL-C were increased in the HFCD-FRB group compared to the HFCD group. The expression of SREBP-1c, fatty acid synthase (FAS), CCAAT-enhancer-binding protein α (C/EBPα), and acetyl CoA carboxylase (ACC) were significantly decreased in the HFCD-FRB group compared to the HFCD group. Significant improvements were also observed in all the parameters in the HFCD-RB group compared to the HFCD group, but the HFCD-FRB group showed a superior result. The study claimed that FRB has anti-obesity properties and could improve lipid metabolism [99].

High-fat-diet-induced obese C57BL/6 male mice were supplemented with RB oil (RBO) or palm oil (PO) (170 g of RBO or PO/Kg of food; no changes in food consumption between groups) for 10 weeks. RBO-supplemented mice reduced epididymal white adipose tissue (EWAT) weights compared to the control. RBO supplementation suppressed the expression of SREBP-1c, PPAR-γ, and M2-macrophage markers (iNOS, COX-2, and f4/80) in mice’s EWAT. Additionally, RBO supplementation favorably altered the expression of surface M2 makers (CD206 and CD11c), arginase 1 (arg1), and chitinase-like proteins (ym1) in EWAT. RBO supplementation improved the anti-inflammatory status (increased the IL-10 level and decreased the IL-6 and TNF-α levels) in bone marrow-derived macrophages. The study showed that RBO could improve obesity-induced chronic inflammation by altering the expression of inflammation-associated markers and macrophage polarization [100].

Yang et al. reported that supplementing 4 and 8% of RB in food significantly improved high-energy diet-induced obesity in rats. In detail, RB supplementation significantly reduced the adipocyte size and body weight, hepatic TC and TG, and serum glucose and uric acid levels compared to the control. Moreover, the RB supplementation improved hepatic lipid homeostasis. The study claimed RB could prevent obesity-associated metabolic diseases [101].

The supplementation of solvent (hexane) extract of IR-64 RB (100 or 150, or 200 mg/kg BW of RBE for 6 weeks) to high-fat-diet-induced obese rats significantly reduced the body weight, TG, and malondialdehyde. The beneficial effects have remained between 150 and 200 mg of RBE supplementation. The study claimed that RBE supplementation has anti-obesity activity, but further investigations are needed to confirm the result [102].

High-fat-diet-fed male ICR mice were supplemented with low (220 mg/kg BW/day) and high doses (1100 mg/kg BW/day) of RBWE for 8 weeks, and changes in blood pressure, inflammatory markers, and hepatic steatosis were analyzed. RBWE supplementation significantly reduced diastolic blood pressure, serum, and liver TNF-*α* and malondialdehyde (MDA) levels, nuclear factor-κB (NF-*κ*B) levels in the liver and heart, lipid accumulation in the liver, myocardial COX-2, and matrix metalloprotease-9 (MMP-9) levels [94]. Moreover, RBWE supplementation significantly reduced the adipose tissue mass and vascular endothelial growth factor (VEGF) and MMP-2 expressions in visceral fat tissue [95]. The study claimed that RBWE could reduce obesity-associated hypertension and adipose tissue mass and improve health status [103,104].

High-fat-diet-fed male Wistar rats were supplemented with anthocyanin-rich black RB extract (BRBE) (100 or 200 mg/kg BW/day) for 8 weeks. BRBE supplementation significantly reduced body weight, visceral fat weight, TC, TG, and plasma glucose levels, and serum creatinine and renal cortical MDA levels in high-fat-diet-fed rats. Additionally, BRBE supplementation attenuates kidney injury by reducing oxidative stress and renal cell damage [105].

High-sugar-diet-fed male Wistar rats were supplemented with 11% RB in their diet for 20 weeks. The RB supplementation significantly reduced body weight, body fat, adiposity index, TG, insulin, Homeostatic Model Assessment for Insulin Resistance (HOMA-IR), pro-inflammatory markers (IL-6, and TNF-α), and oxidative stress (MDA, Superoxide dismutase (SOD), and CAT (Catalase)). RB supplementation significantly improved cardiac dysfunction in high-sugar-diet-fed rats [106].

Multiple strain (*Bacillus amyloliquefaciens* M4, *Bacillus subtilis* M5, *Bacillus* sp.M6, *Lactobacillus casei, Bifidobacterium bifidum,* and *Aspergillus oryzae*)-mediated fermented RB was supplemented to high-fat-diet-fed female C57BL/6J mice (0.239% of FRB in the diet per day) for 8 weeks. The changes in the microbiota and metabolites were reported. FRB supplementation reduced weight gain, the abundance of *Enterococcus* and *Peptostreptococcaceae*, and fecal succinic acid concentration. Moreover, the levels of xylitol, sorbitol, uracil, glutamic acid, and malic acid were decreased, while the fumaric acid level was increased in peripheral blood. FRB supplementation did not increase the abundance of beneficial microbes, but the abundance of the harmful microbial level was decreased. The study showed that FRB supplementation reduced high-fat-diet-induced obesity by modifying gut microbiota and host metabolism [107].

The supplementation of red RB ethanolic extract (RRBEE; 0.5 or 1 g/kg BW/day) for 12 weeks ameliorates the high-fat-diet-induced obesity-associated complications and insulin resistance in male ICR mice. RRBEE supplementation induced the expression of IRS in the adipose tissue and GLUT in the adipose tissue and muscles. At the same time, RRBEE supplementation reduced the expression of IDE in muscles and pancreatic insulin. Moreover, the size of the pancreatic islet was reduced in RRBEE-supplemented mice [98] (Table 2).

The studies indicated that RB extracts improved obesity by regulating cholesterol profiles, host metabolism, and antioxidant, inflammatory, and immune signaling networks. The possible mechanism behind the anti-obesity activity of RB has been detailed in Section 5.

### 3.3. Clinical Studies

The cholesterol-lowering property of RB extract containing acylated steryl glucoside (RB-ASG) has been reported. In detail, obese Japanese men were supplemented with RB-ASG (30–50 mg/day) for 12 weeks, and their anthropometric parameters, pressure, fat, and cholesterol contents were determined. RB-ASG supplementation significantly reduced the TC, LDL-C, non-HDL-C, LDL/HDL ratio, abdominal circumference, and subcutaneous fat area in obese men. No significant changes were observed in systolic and diastolic blood pressure and cholesterol composition. The study showed that the consumption of RB-ASG could improve dyslipidemia in obese subjects [21].

Chinese adults with borderline hypercholesterolemia were supplemented with 30 g of refined olive oil or blended oil 1 (BO1; 8000:720:300 mg/kg of oil of oryzanol: sesamin: sesamolin) and blended oil 2 (BO2; 4800:300:125 mg/kg of oil of oryzanol: sesamin: sesamolin) for 8 weeks. After 8 weeks of intervention, changes in the lipid profile were assessed. TC, LDL-C, TG, HDL-C, apoB-to-apoA1 ratio, blood pressure, and serum glucose levels were reduced significantly in all groups. The body weight of the subjects was also significantly increased in all groups. All the groups had no changes in apoA1 and HDL-C levels after treatment [22].

Overweight and obese adults on a 25% calorie-restricted diet were supplemented with pigmented RB (PRB) or PRB with plant sterols (PRB + PS) (30 g per day) for 8 weeks. After 8 weeks of supplementation, all the participants lost weight, and the weight loss did not significantly differ between the PRB and PRB + PS groups. The PRB + PS group showed significant levels of reduction in TC and LDL-C. PRB or PRB + PS supplementation significantly reduced the blood pressure, serum level of F2-isoprostanes, and leptin compared to baseline. The study claimed that the supplementation of PRB or PRB + PS, along with a calorie-restricted diet, could aid in reducing the risk of cardiovascular disease [23].

Similarly, overweight and obese adults on energy-restriction diets were supplemented with RB (70 g of RB/day) or rice husk (RH) (25 g of RH/day) for 12 weeks. The changes in the inflammatory markers and anthropometric changes were determined. All groups significantly reduced BMI, weight, and waist circumference. There were no significant changes in hs-CRP and IL-6 levels between RB- and RH-supplemented groups. However, significant differences were observed in serum hs-CRP and IL-6 levels in both RB- and RH-treated groups compared to their respective baseline values. The study revealed that supplementing RB or RH and an energy-restriction diet plan could improve the studied inflammatory markers in overweight and obese adults [24] (Table 3). The clinical studies showed that the RB extracts and calorie-restricted diet synergistically reduce obesity. RB with ASG and plant sterols showed better results, which could reduce the risk of cardiovascular disease and dyslipidemia. More clinical studies are needed to define the duration, dose, and combination of other natural supplements and RB supplementation to prevent and manage obesity.

## 4. Influence of Rice Bran Supplementation on Host Microbiome

The impact of RB supplementation on the microbiome of overweight and obese subjects has not been reported in detail. Some recent studies reported RB’s influences on the obese host microbiome [107,108,109,110,111].

*Bacillus amyloliquefaciens* M4, *B. subtilis* M5, *Bacillus* sp. M6, *Lactobacillus casei*, *Bifidobacterium bifidum*, and *Aspergillus oryzae*-mediated fermented RB supplementation improved the microbiome and metabolism in high-fat-induced obese C57BL/6J mice. In detail, mice were fed a high-fat diet and 0.239% fermented RB for 8 weeks. The microbiome analysis showed that fermented RB supplementation did not increase the beneficial microbes, whereas it suppressed the unclassified family *Peptostreptococcaceae* and *Enterococcus.* Moreover, fermented RB supplementation significantly increased the fecal succinic acid concentration, correlated with the unclassified family *Peptostreptococcaceae*, *Turicibacter*, and *Enterococcus* abundances. Blood glutamic acid, malic acid, uracil, xylitol, and sorbitol levels were decreased, while the fumaric acid level was increased in the fermented RB-supplemented mice. The author claimed that fermented RB supplementation could affect the host metabolism and gut microbiome in high-fat-induced obese C57BL/6J mice [107].

Rb (20% in diet) and *Saccharomyces cerevisiae* Misaki-1 and *Lactobacillus plantarum* Sanriku-SU8-mediated fermented RB (FRB; 20% in diet) were supplemented to high sucrose and no-fiber-fed ICR mice for 2 weeks. The FRB supplementation decreased the TG and TC values. The RB-supplemented mice showed higher fecal frequency. Regarding the microbiome, the α-diversity of the microbiome was increased in RB- and FRB-supplemented groups. The abundance of *Bacteroidetes* and *Firmicutes* was increased in RB- and FRB-supplemented groups, respectively. FRB supplementation also reduced the microbes associated with diabetes and gut toxicity. The study claimed that FRB improved the gut microbiome and lipid profile of the high sucrose and no-fiber-fed ICR mice [108].

The influence of RB oil (RBO) on isoflavonoids and intestinal microbiota was studied in mice. Mice were supplemented with daidzein (0.05%) and RBO (10%) in the diet for 30 days. The RBO-supplemented group showed less urine daidzein and dihydrodaidzein, and a higher equol/daidzein ratio and fecal bile acids than the control group. RBO supplementation significantly increased the abundance of *Lactobacillales*, and a positive correlation was observed between fecal bile acids and *Lactobacillales.* The study stated that dietary RBO influences daidzein metabolism by modifying the intestinal microbiome [109].

Zhao et al. reported the antihyperlipidemic effect of RB-hydrolyzed bound phenolics (RB-HBP). C57BL/6J male mice were supplemented with RB-HBP (100 mg/kg/day) for 14 weeks. In high-fat-diet-fed mice, RB-HBP supplementation significantly reduced serum TC, LDL-C, free fatty acid levels, fecal TG, and total bile acid levels. RB-HBP supplementation hindered the activation of sterol regulatory element binding protein 1c (SREBP-1c), peroxisome proliferators-activated receptors-γ (PPARγ), and nuclear receptors liver X receptor-α (LXRα) in mice. Thus, low-density lipoprotein receptor (LDLR), cluster of differentiation 36 (CD36), acyl-CoA carboxylase (ACC1), fatty acid synthase (FAS), and diacylglycerol O-acyltransferase 2 (DGAT2) expressions, both mRNA and protein levels, were altered in mice’s livers. RB-HBP supplementation increased the α-diversity, *Bacteroidetes/Firmicutes* ratio, *Bacteroides, Allobaculum, Rikenellaceae_RC9_gut_group,* and *Faecalibaculum*. It suppressed the abundance of *Alistipes, Odoribacter, Butyricimonas, Parabacteroides, unclassified_f_Lachnospiraceae*, *Ruminiclostridium_9, Romboutsia,* and *norank_f_Erysipelotrichaceae* in high-fat-diet-fed mice. The results indicated that RB-HBP supplementation could improve the lipid profile by regulating lipogenesis, cholesterol absorption, and gut microbiome [110].

The supplementation of RRB (Raw RB), RRBS (RRB stored for 3 months), IRRB (Infrared radiation-stabilized RB), and IRRBS (IRRB stored for 3 months) (300 mg/kg BW/day for 39 days) improved the obesity-associated parameters and gut microbiota in high-fat-diet-fed C57BL/6 mice. The RB supplementation reduced the body weight gain, serum TC, TG, LDL-C, alanine aminotransferase, aspartate aminotransferase levels, and TNF-α, IL-6, and INF-γ. The level of HDL-C/TC increased in RB-supplemented groups. The RB supplementation decreased the expressions of uncoupling protein 1 (UCP1), positive regulatory domain containing 16 (PRDM16), and peroxisome proliferator-activated receptor gamma coactivator 1-alpha (PGC-1α) and increased the expressions of transcription factor 21 (TCF21) and homeobox protein Hox-C8 (HOXC8). The RB supplementation attenuated liver fat accumulation and enhanced white adipose browning, except for RRBS. The microbiome analysis showed that RB supplementation improved the abundance of *Bacteroidetes* and the *Bacteroidetes*/*Firmicutes* ratio. Moreover, the abundance of *Desulfovibrio* was decreased, while *Akkermansia* and *Lachnospiraceae* abundance was increased in RB-supplemented groups. These changes were not observed in the RRBS-supplemented group. The results indicated that RRB, IRRB, and IRRBS potently improved the lipid profile, inflammatory status, and microbiome of the high-fat-diet-fed obese mice model [111].

The studies revealed that RB supplementation aids in normalizing the host microbiome by reducing the abundance of pathogens and supporting the growth of beneficiary microbes. RB derivatives could act as prebiotics that could promote healthy normal microbial flora. The healthy microbial composition provides bioactive metabolites, further improving the host’s health.

Several studies reported RB or RB derivatives-mediated changes in the host microbiome (Table 4), other than in obese conditions [112,113,114,115,116,117,118,119,120]. For example, arabinoxylan (AX) is found in RB, and the prebiotic property of AX has been studied in vitro. The in vitro fecal fermentation study with AX showed that it modified the fecal microbiota of obese and non-obese subjects. *Collinsella*, *Blautia*, and *Bifidobacterium* were increased; *Sutterella*, *Bilophila*, and *Parabacteroides* were decreased. Moreover, AX significantly increased the total and individual short-chain fatty acid levels [121].

## 5. Mechanisms Associated with the Anti-Obesity Property of Rice Bran

Studies in human and rodent models have suggested that dietary RB improves the cholesterol profile, antioxidant and inflammatory status, blood and vascular parameters, adipose, liver, and pancreas parameters, host metabolism, and microbiome, thereby delivering health benefits to the host.

The anti-obesity property of RB was not attributed to a single biological event, which is the result of several bioprocesses. The complete molecular mechanism associated with the beneficial effects of dietary RB has yet to be elucidated.

Briefly, RB supplementation alters the host metabolism (SCFAs, serum metabolites) [107] and facilitates fecal cholesterol excretion [86].

The overall host inflammatory system was improved during RB supplementation. In particular, C-RP, TNF-α, COX-2, VCAM-1, IL-6, and IL-1β levels were improved in serum and adipose tissue and improved vascular and microvascular inflammation [86,87,89,100]. Moreover, RB supplementation improved the expression of IRS-2 (insulin receptor), GLUT-2 (glucose transporter), and glucokinase in the pancreas [96], glucose–insulin homeostasis [97], and reduced the pancreatic islet size and pancreatic insulin [98].

Obesity alters adipocytes and several other cellular mechanisms, which causes an increase in systemic oxidative stress [120] and chronic inflammation [122]. Dietary RB improved the antioxidant system of the host by significantly affecting the levels and expressions of iNOS, superoxide, NADPH oxidase, and SOD and CAT activities [86,90,108].

RB intervention significantly improved adipogenesis, reduced adipocyte size and adipose tissue mass, reduced adipocyte hypertrophy and lipid accumulation, and expressions of VEGF and MMP-2 in visceral fat tissue [89,93,97,103,104].

**Figure 2:**
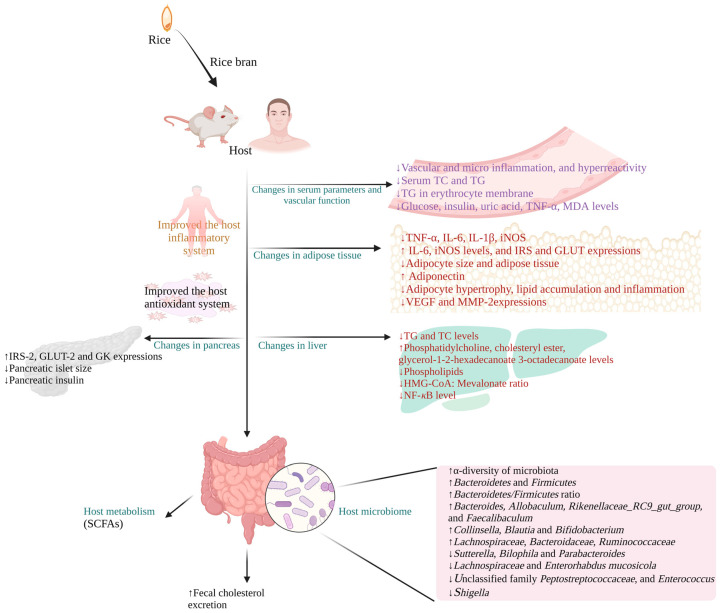
The possible mechanism underlying the anti-obese property of rice bran. Rice bran improves the host inflammatory and antioxidant systems and positively regulates the liver, pancreas, vascular functions, host metabolism, and host microbiome. Moreover, rice bran supplementation facilitates fecal cholesterol excretion. TC: Total cholesterol; TG: Triglycerides; TNF-α: Tumor necrosis factor-α; MDA: Malondialdehyde; IL-6: Interleukin-6; IL-1β: Interleukin-1β; iNOS: Inducible nitric oxide synthase; IRS: Insulin receptor substrate; GLUT: Glucose transporter; VEGF: vascular endothelial growth factor; MMP-2: Matrix metalloprotease-2; HMG-CoA: 3-hydroxy-3-methylglutaryl coenzyme A; NF-κB: Nuclear factor- κB; GK: Glucokinase; SCFAs: Short-chain fatty acids; ↓: Decrease; ↑: Increase.

Regarding the microbiome, dietary RB and fermented RB significantly improved the beneficial microbial load (*Bacteroidetes* and *Firmicutes*) and α-diversity of microbiota and reduced the pathogenic bacterial load (e.g., *Shigella* sp.) in obese experimental models [109,110,112]. The possible comprehensive mechanisms relating to the anti-obesity property of RB have been illustrated (Figure 2).

## 6. Conclusions

The RB composition varies on several factors, including cultivars and geographic and weather conditions during the cultivation. Preclinical studies showed that the RB extracts, RB fractions, γ-oryzanol, and fermented RB supplementation improved vascular function, liver, and pancreas states, reduced adipocyte hypertrophy and lipid accumulation, improved the host antioxidant and inflammatory system, and host metabolism, and increased fecal cholesterol excretion. Specifically, dietary RB and its fractions improved the host microbiota and aided in restoring eubiosis. Because of the above-mentioned impact on the host, dietary RB collectively acts as an anti-obesity therapeutic agent. However, the therapeutic nature of RB needed to be developed appropriately. Further studies on dosage, duration of the supplementation, therapeutic strategies, and pharmacological and pharmacokinetic properties of dietary RB are mandatory. Developing RB-based functional products and their daily utilization may help prevent and manage obesity.

## Figures and Tables

**Table 1:** Details of the phytochemical composition of rice bran (RB) of different rice varieties.

Cultivar/Strain	Phytochemical Contents	Extraction Methods/Method of Analysis	Ref.
Hashemi RB	TPC: 221.06 ± 10.63 mg/100 g DM TFC: 108.50 ± 10.01 mg/100 g DM Total tocopherol: 38.11 ± 2.04 mg/100 g DM Total tocotrienol 46.54 ± 2.92 mg/100 g DM	Ethanol maceration	[27]
	TPC: 270.51 ± 11.47 mg/100 g DM TFC: 137.15 ± 12.89 mg/100 g DM Total tocopherol: 36.93 ± 2.26 mg/100 g DM Total tocotrienol: 55.83 ± 1.85 mg/100 g DM	Ethanol–water (50:50) maceration	
	TPC: 246.34 ± 12.26 mg/100 g DM TFC: 112.60 ± 13.65 mg/100 g DM Total tocopherol: 37.08 ± 2.21 mg/100 g DM Total tocotrienol: 51.28 ± 2.80 mg/100 g DM	Ethanol ultrasonic	
	TPC: 288.40 ± 14.35 mg/100 g DM TFC: 156.20 ± 10.69 mg/100 g DM Total Tocopherol: 37.51 ± 2.05 mg/100 g DM Total Tocotrienol: 56.23 ± 2.37 mg/100 g DM	Ethanol–water (50:50) ultrasonic	
KDML105	γ-oryzanol: 171.23 ± 0.16 mg/100 g CF α-tocopherol: 6.62 ± 0.01 mg/100 g CF β-tocopherol: 0.38 ± 0.00 mg/100 g CF γ-tocopherol: 7.91 ± 0.00 mg/100 g CF δ-tocopherol: 0.13 ± 0.01 mg/100 g CF Gallic acid: 0.09 ± 0.01 mg/100 g sample ^1^ Caffeic acid: 0.17 ± 0.00 mg/100 g sample ^1^ Epigallocatechin gallate: 0.42 ± 0.09 mg/100 g sample ^1^ *p*-coumaric acid: 0.36 ± 0.01 mg/100 g sample ^1^ *o*-coumaric acid: 0.57 ± 0.04 mg/100 g sample ^1^ Quercetin: 0.27 ± 0.04 mg/100 g sample ^1^ Ferulic acid: 0.17 ± 0.01 mg/100 g sample ^1^	HPLC-Mass spectrometry	[40]
BB3 CMU	γ-oryzanol: 219.90 ± 0.12 mg/100 g CF α-tocopherol: 10.37 ± 0.04 mg/100 g CF β-tocopherol: 0.58 ± 0.00 mg/100 g CF γ-tocopherol: 6.13 ± 0.02 mg/100 g CF δ-tocopherol: 0.18 ± 0.00 mg/100 g CF Gallic acid: 0.14 ± 0.00 mg/100 g sample ^1^ Caffeic acid: 0.30 ± 0.01 mg/100 g sample ^1^ Epigallocatechin gallate: 1.34 ± 0.06 mg/100 g sample ^1^ *p*-coumaric acid: 1.15 ± 0.07 mg/100 g sample ^1^ *o*-coumaric acid: 0.61 ± 0.17 mg/100 g sample ^1^ Quercetin: 0.26 ± 0.00 mg/100 g sample ^1^ Ferulic acid: 0.18 ± 0.00 mg/100 g sample ^1^		
BB4 CMU	γ-oryzanol: 220.43 ± 0.09 mg/100 g CF α-tocopherol: 15.84 ± 0.03 mg/100 g CF β-tocopherol: 1.16 ± 0.01 mg/100 g CF γ-tocopherol: 6.78 ± 0.04 mg/100 g CF δ-tocopherol: 0.21 ± 0.01 mg/100 g CF Gallic acid: 0.15 ± 0.00 mg/100 g sample ^1^ Caffeic acid: 0.31 ± 0.00 mg/100 g sample ^1^ Epigallocatechin gallate: 0.96 ± 0.04 mg/100 g sample ^1^ *p*-coumaric acid: 0.87 ± 0.01 mg/100 g sample ^1^ *o*-coumaric acid: 0.57 ± 0.01 mg/100 g sample ^1^ Quercetin: 0.24 ± 0.01 mg/100 g sample ^1^ Ferulic acid: 0.17 ± 0.01 mg/100 g sample ^1^		
RD6	γ-oryzanol: 207.79 ± 0.03 mg/100 g CF α-tocopherol: 9.22 ± 0.06 mg/100 g CF β-tocopherol: 0.27 ± 0.01 mg/100 g CF γ-tocopherol: 9.19 ± 0.04 mg/100 g CF δ-tocopherol: 0.13 ± 0.00 mg/100 g CF Gallic acid: 0.13 ± 0.00 mg/100 g sample ^1^ Caffeic acid: 0.19 ± 0.00 mg/100 g sample ^1^ Epigallocatechin gallate: 1.21 ± 0.02 mg/100 g sample ^1^ *p*-coumaric acid: 0.41 ± 0.02 mg/100 g sample ^1^ *o*-coumaric acid: 0.51 ± 0.00 mg/100 g sample ^1^ Quercetin: 0.17 ± 0.00 mg/100 g sample ^1^ Ferulic acid: 0.21 ± 0.01 mg/100 g sample ^1^		
KC CMU107	γ-oryzanol: 218.76 ± 0.13 mg/100 g CF α-tocopherol: 4.80 ± 0.02 mg/100 g CF β-tocopherol: 0.62 ± 0.00 mg/100 g CF γ-tocopherol: 6.28 ± 0.01 mg/100 g CF δ-tocopherol: 0.11 ± 0.00 mg/100 g CF CY 3-GLU: 40.61 ± 0.39 mg/100 g sample ^1^ PN 3-GLU: 15.72 ± 0.13 mg/100 g sample ^1^ Caffeic acid: 0.12 ± 0.00 mg/100 g sample ^1^ Epigallocatechin gallate: 0.32 ± 0.00 mg/100 g sample ^1^ *p*-coumaric acid: 0.22 ± 0.01 mg/100 g sample ^1^ *o*-coumaric acid: 0.15 ± 0.00 mg/100 g sample ^1^ Quercetin: 1.22 ± 0.01 mg/100 g sample ^1^ Ferulic acid: 0.33 ± 0.00 mg/100 g sample ^1^		
BKU5 CMU	γ-oryzanol: 145.16 ± 0.06 mg/100 g CF α-tocopherol: 15.20 ± 0.01 mg/100 g CF β-tocopherol: 0.77 ± 0.00 mg/100 g CF γ-tocopherol: 4.46 ± 0.02 mg/100 g CF δ-tocopherol: 0.18 ± 0.00 mg/100 g CF CY 3-GLU: 331.10 ± 1.64 mg/100 g sample ^1^ PN 3-GLU: 34.26 ± 0.23 mg/100 g sample ^1^ Caffeic acid: 0.40 ± 0.02 mg/100 g sample ^1^ Epigallocatechin gallate: 0.47 ± 0.01 mg/100 g sample ^1^ *p*-coumaric acid: 0.46 ± 0.01 mg/100 g sample ^1^ *o*-coumaric acid: 0.05 ± 0.03 mg/100 g sample ^1^ Quercetin: 1.15 ± 0.01 mg/100 g sample ^1^ Ferulic acid: 0.28 ± 0.00 mg/100 g sample ^1^		
K4 CMU	γ-oryzanol: 228.96 ± 0.02 mg/100 g CF α-tocopherol: 6.39 ± 2.77 mg/100 g CF β-tocopherol: 0.47 ± 0.95 mg/100 g CF γ-tocopherol: 8.51 ± 2.92 mg/100 g CF δ-tocopherol: 0.36 ± 0.25 mg/100 g CF Epigallocatechin gallate: 0.68 ± 0.02 mg/100 g sample ^1^ *p*-coumaric acid: 0.44 ± 0.01 mg/100 g sample ^1^ *o*-coumaric acid: 0.25 ± 0.02 mg/100 g sample ^1^ Quercetin: 1.26 ± 0.02 mg/100 g sample ^1^ Ferulic acid: 0.20 ± 0.01 mg/100 g s sample ^1^		
KDK	γ-oryzanol: 222.34 ± 0.25 mg/100 g CF α-tocopherol: 5.65 ± 0.04 mg/100 g CF β-tocopherol: 0.97 ± 0.00 mg/100 g CF γ-tocopherol: 6.81 ± 0.02 mg/100 g CF δ-tocopherol: 0.22 ± 0.00 mg/100 g CF CY 3-GLU: 41.86 ± 0.12 mg/100 g sample ^1^ PN 3-GLU: 23.75 ± 0.68 mg/100 g sample ^1^ Caffeic acid: 0.09 ± 0.00 mg/100 g sample ^1^ Epigallocatechin gallate: 0.28 ± 0.01 mg/100 g sample ^1^ *p*-coumaric acid: 0.15 ± 0.00 mg/100 g sample ^1^ *o*-coumaric acid: 0.18 ± 0.04 mg/100 g sample ^1^ Quercetin: 0.81 ± 0.00 mg/100 g sample ^1^ Ferulic acid: 0.13 ± 0.00 mg/100 g sample ^1^		
KAK1 CMU	γ-oryzanol: 175.74 ± 0.25 mg/100 g CF α-tocopherol: 17.56 ± 0.00 mg/100 g CF β-tocopherol: 0.87 ± 0.01 mg/100 g CF γ-tocopherol: 4.44 ± 0.02 mg/100 g CF δ-tocopherol: 0.17 ± 0.00 mg/100 g CF CY 3-GLU: 525.72 ± 1.72 mg/100 g sample ^1^ PN 3-GLU: 46.01 ± 0.51 mg/100 g sample ^1^ Caffeic acid: 0.15 ± 0.00 mg/100 g sample ^1^ Epigallocatechin gallate: 0.50 ± 0.00 mg/100 sample ^1^ *p*-coumaric acid: 0.33 ± 0.01 mg/100 g sample ^1^ *o*-coumaric acid: 0.33 ± 0.02 mg/100 g sample ^1^ Quercetin: 1.21 ± 0.01 mg/100 g sample ^1^ Ferulic acid: 0.23 ± 0.01 mg/100 g sample ^1^		
Sang5 CMU	γ-oryzanol: 111.36 ± 0.22 mg/100 g CF α-tocopherol: 4.77 ± 0.06 mg/100 g CF β-tocopherol: 0.64 ± 0.00 mg/100 g CF γ-tocopherol: 6.02 ± 0.04 mg/100 g CF δ-tocopherol: 0.18 ± 0.00 mg/100 g CF CY 3-GLU: 166.40 ± 0.57 mg/100 g sample ^1^ PN 3-GLU: 13.09 ± 0.01 mg/100 g sample ^1^ Caffeic acid: 0.16 ± 0.01 mg/100 g sample ^1^ Epigallocatechin gallate: 0.42 ± 0.03 mg/100 g sample ^1^ *p*-coumaric acid: 0.23 ± 0.04 mg/100 g sample ^1^ *o*-coumaric acid: 0.55 ± 0.03 mg/100 g sample ^1^ Quercetin: 1.27 ± 0.01 mg/100 g sample ^1^ Ferulic acid: 0.22 ± 0.00 mg/100 g sample ^1^		
PES1 CMU	γ-oryzanol: 139.58 ± 0.04 mg/100 g CF α-tocopherol: 17.02 ± 0.03 mg/100 g CF β-tocopherol: 0.72 ± 0.00 mg/100 g CF γ-tocopherol: 4.35 ± 0.03 mg/100 g CF δ-tocopherol: 0.25 ± 0.00 mg/100 g CF CY 3-GLU: 650.55 ± 1.65 mg/100 g sample ^1^ PN 3-GLU: 67.54 ± 0.32 mg/100 g sample ^1^ Caffeic acid: 0.26 ± 0.00 mg/100 g sample ^1^ Epigallocatechin gallate: 0.39 ± 0.00 mg/100 g sample ^1^ *p*-coumaric acid: 0.26 ± 0.00 mg/100 g sample ^1^ *o*-coumaric acid: 0.39 ± 0.01 mg/100 g sample ^1^ Quercetin: 1.44 ± 0.00 mg/100 g sample ^1^ Ferulic acid: 0.24 ± 0.01 mg/100 g sample ^1^		
Taibalang black waxy rice	Outer RB: Total ACN: 6.29 ± 0.08 mg CY 3-GLU Eq/g DM CY 3-GLU: 2.44 ± 0.27 mg/g DM PN 3-GLU: 0.53 ± 0.04 mg/g DM CY 3-RUT: 0.46 ± 0.04 mg/g DM Vitamin E total: 85.49 ± 3.24 µg/g DM γ-oryzanol: 3.95 ± 0.32 mg/g DM Inner RB: Total ACN: 3.46 ± 0.11 mg CY 3-GLU Eq/g DM CY 3-GLU: 1.43 ± 0.19 mg/g DM PN 3-GLU: 0.34 ± 0.05 mg/g DM CY 3-RUT: 0.25 ± 0.04 mg/g DM	80% ethanol extraction and HPLC analysis	[63]
Black rice western Taiwan	Outer RB: Total ACN: 6.70 ± 0.06 mg CY 3-GLU Eq/g DM CY 3-GLU: 3.07 ± 0.14 mg/g DM PN 3-GLU: 1.32 ± 0.03 mg/g DM CY 3-RUT: 0.42 ± 0.05 mg/g DM Vitamin E total: 129.97 ± 1.23 µg/g DM γ-oryzanol: 4.85 ± 0.11 mg/g DM Inner RB: Total ACN: 4.92 ± 0.30 mg CY 3-GLU Eq/g DM CY 3-GLU: 2.06 ± 0.18 mg/g DM PN 3-GLU: 0.89 ± 0.07 mg/g DM CY 3-RUT: 0.28 ± 0.05 mg/g DM		
Black rice Thailand	Outer RB: Total ACN: 11.27 ± 0.38 mg CY 3-GLU Eq/g DM CY 3-GLU: 10.63 ± 0.66 mg/g DM PN 3-GLU: 0.81 ± 0.06 mg/g DM CY 3-RUT: 0.52 ± 0.12 mg/g DM Vitamin E total: 137.28 ± 9.75 µg/g DM γ-oryzanol: 7.72 ± 0.39 mg/g DM Inner RB: Total ACN: 6.85 ± 0.36 mg CY 3-GLU Eq/g DM CY 3-GLU: 6.62 ± 0.39 mg/g DM PN 3-GLU: 0.40 ± 0.05 mg/g DM CY 3-RUT: 0.36 ± 0.11 mg/g DM		
Taibalang red waxy rice	Outer RB: Pro-ACN: 19.13 ± 0.41 mg CE/g DM Total ACN: 0.31 ± 0.01 mg CY 3-GLU Eq/g DM CY 3-GLU: 0.05 ± 0.01 mg/g DM Vitamin E total: 99.68 ± 9.14 µg/g DM γ-oryzanol: 3.62 ± 0.16 mg/g DM Inner RB: Pro-ACN: 3.41 ± 0.08 mg CE/g DM Total ACN: 0.20 ± 0.01 mg CY 3-GLU Eq/g DM CY 3-GLU: 0.03 ± 0.01 mg/g DM		
Guangfu red rice	Outer RB: Pro-ACN: 17.41 ± 0.63 mg CE/g DM Total ACN: 0.38 ± 0.03 mg CY 3-GLU Eq/g DM CY 3-GLU: 0.20 ± 0.04 mg/g DM Vitamin E total: 166.93 ± 3.65 µg/g DM γ-oryzanol: 3.59 ± 0.23 mg/g DM Inner RB: Pro-ACN: 4.31 ± 0.77 mg CE/g DM Total ACN: 0.22 ± 0.01 mg CY 3-GLU Eq/g DM CY 3-GLU: 0.07 ± 0.01 mg/g DM		
Red rice Thailand	Outer RB: Pro-ACN: 12.16 ± 0.43 mg CE/g DM Total ACN: 0.35 ± 0.02 mg CY 3-GLU Eq/g DM CY 3-GLU: 0.15 ± 0.04 mg/g DM PN 3-GLU: 0.03 ± 0.00 mg/g DM Vitamin E total: 50.65 ± 5.07 µg/g DM γ-oryzanol: 3.69 ± 1.07 mg/g DM Inner RB: Pro-ACN: 0.75 ± 0.40 mg CE/g DM Total ACN: 0.28 ± 0.01 mg CY 3-GLU Eq/g DM CY 3-GLU: 0.08 ± 0.02 mg/g DM PN 3-GLU: 0.01 ± 0.00 mg/g DM		
RB of KDML105	TPC: Raw: 3.52 ± 0.06 mg GAE/g DW Hot air: 3.58 ± 0.03 mg GAE/g DW FIR: 4.05 ± 0.03 mg GAE/g DW Cellulase: 3.05 ± 0.03 mg GAE/g DW TFC: Raw: 3.88 ± 0.09 mg RE/g DW Hot air: 3.08 ± 0.10 mg RE/g DW FIR: 3.59 ± 0.16 mg RE/g DW Cellulase: 3.72 ± 0.10 mg RE/g DW γ-Oryzanol: Raw: 5.701 ± 0.022 mg/g of RFRB Hot air: 5.281 ± 0.018 mg/g of RFRB FIR: 5.612 ± 0.006 mg/g of RFRB Cellulase: 5.698 ± 0.012 mg/g of RFRB α-Tocopherol: Raw: 82.15 ± 2.84 µg/g of RFRB Hot air: 63.50 ± 2.56 µg/g of RFRB FIR: 95.78 ± 3.81 µg/g of RFRB Cellulase: 83.42 ± 5.26 µg/g of RFRB γ-Tocopherol: Raw: 5.04 ± 0.02 µg/g of RFRB Hot air: 5.03 ± 0.07 µg/g of RFRB FIR: 5.14 ± 0.09 µg/g of RFRB Cellulase: 5.04 ± 0.03 µg/g of RFRB δ-Tocopherol FIR: 7.84 ± 0.12 µg/g of RFRB	Hot air, far-infrared radiation, cellulase treatment.	[75]
IR64	Oryzanol: Control: 267.3 ± 0.75 mg/100 g of RB Cellulase: 276.8 ± 0.49 mg/100 g of RB Xylanase: 286.3 ± 1.34 mg/100 g of RB Cellulase + Xylanase: 299.2 ± 1.14 mg/100 g of RB Flavonoid: Control: 32.2 ± 0.61 mg/100 g of RB Cellulase: 40.5 ± 0.50 mg CE/100 g of RB Xylanase: 38.1 ± 0.61 mg CE/100 g of RB Cellulase + Xylanase: 44.4 ± 0.61 mg CE/100 g of RB Soluble Polyphenol: Control: 278 ± 14 mg FA/100 g of RB Cellulase: 314 ± 6 mg FA/100 g of RB Xylanase: 304 ± 4 mg FA/100 g of RB Cellulase + Xylanase: 324 ± 3 mg FA/100 g Bound Polyphenol: Control: 255 ± 17 mg FA/100 g of RB Cellulase: 272 ± 4 mg FA/100 g of RB Xylanase: 305 ± 4 mg FA/100 g of RB Cellulase + Xylanase: 285 ± 2 mg FA/100 g of RB	For oryzanol extraction: Petroleum ether For soluble and bound polyphenol extraction: Methanol in 1% HCL. For flavonoid extraction: Petroleum ether and 1% HCl methanol	[76]
Jyothi	Oryzanol: Control: 159.24 ± 1.13 mg/100 g of RB Cellulase: 164.04 ± 0.49 mg/100 g of RB Xylanase: 167.01 ± 0.95 mg/100 g of RB Cellulase + Xylanase: 174.45 ± 1.31 mg/100 g of RB Flavonoid: Control: 113.7 ± 0.60 mg/100 g of RB Cellulase: 119.3 ± 0.60 mg CE/100 g of RB Xylanase: 116.8 ± 0.61 mg CE/100 g of RB Cellulase + Xylanase: 127.7 ± 0.61 mg CE/100 g of RB Soluble Polyphenol: Control: 502.5 ± 13 mg FA/100 g of RB Cellulase: 741.5 ± 11 mg FA/100 g of RB Xylanase: 752.5 ± 22 mg FA/100 g of RB Cellulase + Xylanase: 794.9 ± 05 mg FA/100 g of RB Bound Polyphenol: Control: 1451 ± 19 mg FA/100 g of RB Cellulase: 1534 ± 16 mg FA/100 g of RB Xylanase: 1588 ± 15 mg FA/100 g of RB Cellulase + Xylanase: 1567 ± 15 mg FA/100 g of RB		
DML105	γ-Oryzanol: Microwave 60 °C: 8.94 mg/g of DRB; 90 °C: 9.08 mg/g of DRB; 110 °C: 8.82 mg/g of DRB Hot air 70 °C: 9.26 mg/g of DRB; 100 °C: 8.93 mg/g of DRB; 180 °C: 8.82 mg/g of DRB. Roasting 60 °C: 8.81 mg/g of DRB; 80 °C: 9.10 mg/g of DRB Parboiling 70 °C: 9.76 mg/g of DRB. Autoclave 121 °C: 8.86 mg/g of DRB. Enzyme 50 °C: 8.31 mg/g of DRB.	Maceration method with pre-treatment processes such as microwave heating, hot air heating, roasting, parboiling, autoclave heating, and enzyme.	[77]
RB of Khao Bahn Nah and Thai jasmine	Tocopherol: Khao Bahn Nah blank: 3.69 ± 0.29 mg/L of RBE Khao Bahn Nah with SSF by *L. casei:* 8.75 ± 1.11 mg/L of RBE Khao Bahn Nah with SSF by *L. plantarum:* 4.09 ± 0.17 mg/L of RBE Thai jasmine blank: 3.35 ± 0.97 mg/L of RBE Thai jasmine with SSF by *L. casei*: 4.51 ± 0.38 mg/L of RBE Thai jasmine with SSF by *L. plantarum:* 7.10 ± 0.23 mg/L of RBE γ-Oryzanol: Khao Bahn Nah blank: 1.55 ± 0.74 mg/L of RBE Khao Bahn Nah with SSF by *L. casei:* 2.57 ± 0.56 mg/L of RBE Khao Bahn Nah with SSF by *L. plantarum:* 1.44 ± 0.36 mg/L of RBE Thai jasmine blank: 1.69 ± 0.35 mg/L of RBE Thai jasmine with SSF by *L. casei*: 3.16 ± 0.15 mg/L of RBE Thai jasmine with SSF by *L. plantarum:* 2.31 ± 0.65 mg/L of RBE Coumaric acid: Thai jasmine with SSF by *L. casei*: 14.47 ± 1.20 mg/L of RBE Ferulic acid: Khao Bahn Nah blank: 18.91 ± 0.60 mg/L of RBE Khao Bahn Nah with SSF by *L. casei:* 30.93 ± 0.81 mg/L of RBE Khao Bahn Nah with SSF by *L. plantarum:* 19.39 ± 0.56 mg/L of RBE Thai jasmine blank: 18.86 ± 1.05 mg/L of RBE Thai jasmine with SSF by *L. casei*: 35.23 ± 0.82 mg/L of RBE Thai jasmine with SSF by *L. plantarum:* 21.61 ± 0.66 mg/L of RBE	Solid state fermentation by *Lactobacillus casei* TISTR 1463 and *Lactobacillus plantarum* TISTR 1465. HPLC analysis of the phenolic compounds.	[78]
RB	γ-Oryzanol: Unfermented: 954.47 ± 21.23 µg/mL of RBE Fermented with *P. acidilactici*: 1148.38 ± 48.20 µg/mL of RBE Fermented with *L. lactis*: 522.26 ± 59.11 µg/mL of RBE Fermented with *P. pentoseous*: 761.82 ± 22.10 µg/mL of RBE α-Tocopherol: Unfermented: 92.25 ± 10.06 µg/mL of RBE Fermented with *P. acidilactici*: 182.37 ± 20.02 µg/mL of RBE Fermented with *L. lactis*: 138.37 ± 15.89 µg/mL of RBE Fermented with *P. pentoseous*: 135.60 ± 12.45 µg/mL of RBE Ferulic acid: Unfermented: 6.19 ± 0.75 µg/mL of RBE Fermented with *P. acidilactici*: 8.56 ± 0.99 µg/mL of RBE Fermented with *P. pentoseous*: 6.96 ± 0.76 µg/mL of RBE Coumaric acid: Unfermented: 10.77 ± 0.52 µg/mL of RBE Fermented with *P. acidilactici*: 3.51 ± 0.14 µg/mL of RBE Fermented with *L. lactis*: 7.29 ± 0.21 µg/mL of RBE Fermented with *P. pentoseous*: 3.79 ± 0.24 µg/mL of RBE TPC: Unfermented: 212.5 ± 0.7 µg GAE/mL of RBE Fermented with *P. acidilactici*: 246 ± 8.5 µg GAE/mL of RBE Fermented with *L. lactis*: 214 ± 16.3 µg GAE/mL of RBE Fermented with *P. pentoseous*: 230 ± 15.6 µg GAE/mL of RBE	Rice bran fermented with *Pediococcus acidilactici, Lactococcus lactis*, and *Pediococcus pentoseous* at 30 °C for 48 h.	[79]
Kum Akha 1′s RB extracts	TPC: 341.31 ± 6.88 mg GAE/g of RBE TFC: 155.21 ± 3.53 mg CAE/g of RBE TA: 132.43 ± 1.69 mg/g of RBE CY 3-GLU: 106.76 ± 2.94 mg/g of RBE PN 3-GLU: 14.48 ± 0.40 mg/g of RBE	Ethanol extraction/ Total flavonoid assay/Total phenolic assay/Total anthocyanin assay and quantification by HPLC	[80]
Sang 5	TPC: 280.56 ± 4.49 mg GAE/g of RBE TFC: 132.39 ± 5.79 mg CAE/g of RBE TA: 126.19 ± 3.36 mg/g of RBE CY 3-GLU: 80.47 ± 5.49 mg/g of RBE PN 3-GLU: 11.42 ± 0.69 mg/g of RBE		
Pieisu 1	TPC: 306.25 ± 2.05 mg GAE/g of RBE TFC: 138.43 ± 3.62 mg CAE/g of RBE TA: 124.61 ± 1.43 mg/g of RBE CY 3-GLU: 100.68 ± 2.17 mg/g of RBE PN 3-GLU: 13.07 ± 0.80 mg/g of RBE		
Kum Doi Saket	TPC: 290.12 ± 1.23 mg GAE/g of RBE TFC: 119.39 ± 3.53 mg CAE/g of RBE TA: 98.63 ± 9.86 mg/g of RBE CY 3-GLU: 40.53 ± 3.12 mg/g of RBE PN 3-GLU: 32.37 ± 1.10 mg/g of RBE		
Kum Chao Morchor 107	TPC: 166.19 ± 3.75 mg GAE/g of RBE TFC: 79.76 ± 2.95 mg CAE/g of RBE TA: 22.32 ± 3.82 mg/g of RBE CY 3-GLU: 4.61 ± 0.10 mg/g of RBE PN 3-GLU: 6.40 ± 0.22 mg/g of RBE		
Bien Koo 5	TPC: 306.34 ± 11.15 mg GAE/g of RBE TFC: 133.35 ± 6.94 mg CAE/g of RBE TA: 114.19 ± 2.04 mg/g of RBE CY 3-GLU: 89.68 ± 6.60 mg/g of RBE PN 3-GLU: 13.21 ± 0.72 mg/g of RBE		
K2	TPC: 157.12 ± 5.67 mg GAE/g of RBE TFC: 75.76 ± 1.72 mg CAE/g of RBE TA: 35.49 ± 5.09 mg/g of RBE CY 3-GLU: 6.84 ± 0.07 mg/g of RBE PN 3-GLU: 6.67 ± 0.25 mg/g of RBE		
K4	TPC: 174.42 ± 1.64 mg GAE/g of RBE TFC: 77.33 ± 7.18 mg CAE/g of RBE TA: 50.57 ± 3.55 mg/g of RBE CY 3-GLU: 8.40 ± 0.19 mg/g of RBE PN 3-GLU: 7.83 ± 0.15 mg/g of RBE		

All values are mean ± SD. RB: Rice bran; TPC: total phenolic content; TFC: Total flavonoid content; DM: Dry material; DW: Dry weight; CF: Crude fat; GAE: Gallic acid equivalent; RE: Rutin equivalent; CE: Catechin equivalent; FA: Ferulic acid; LAB: Lactic acid bacteria; CAE: Caffeic acid equivalent; SSF: Solid state fermentation; TA: Total anthocyanin; CY 3-GLU: Cyanidin-3-glucoside; PN 3-GLU: Peonidin-3-glucoside; CY 3-RUT: Cyanidin-3-rutonoside; FIR: Far-infrared radiation; HPLC- High-performance liquid chromatography; Pro ACN: proanthocyanin; Total ACN: total anthocyanin; Eq: Equivalent; Sample ^1^: Rice bran samples obtained from Lanna Rice Research Center, Chiang Mai University, Chiang Mai, Thailand; RFRB: Rice fractions of rice bran; DRB: Dried rice bran; RBE: Rice bran extract.

**Table 2:** Effects of rice bran supplementation on obesity-associated biomarkers: Results of in vivo studies.

Model	Intervention	Dose and Duration	Results	Ref.
Male Wistar rats	DAG-enriched RB oil (20 and 40%)	10% in the diet for 12 weeks	↓ Serum TG, TC, and BF ↑ Fecal cholesterol excretion ↓ C-RP, TNF-α, platelet aggregation ↓ Expression of iNOS, COX-2, and VCAM-1	[86]
Obese Zucker rats	RB enzymatic extract (RBEE)	1% or 5% RBEE in the diet for 20 weeks	↓ TNF-α, IL-6, IL-1β, iNOS in visceral abdominal adipose tissue. ↑ IL-6 and iNOS in visceral epididymal adipose tissue ↓ Adipocyte size	[87]
Obese Zucker rats	RBEE	1% or 5% RBEE in the diet for 20 weeks	↓ Vascular hyperreactivity ↑eNOS ↓ Vascular inflammation (iNOS, TNF-α) ↓ Superoxide, and NADPH oxidase subunits	[88]
C57BL/6J mice	RBEE	1% or 5% RBEE in the diet for 20 weeks	↓ Insulin resistance Improved the TG, TC, glucose, insulin, adiponectin, and nitrates levels ↓ Adipocyte size ↓ IL-6, and IL-1β in WAT Improved the PPARγ, TNF-α, and Emr1 levels in WAT	[90]
Male albino rats	Diacylglycerol-rich rice bran oil	28 days	↓ TC, non-HDL-C in plasma ↓ TL, TC, TG, and phospholipids in the mesentery ↓ TC, TG, and phospholipids in the liver ↓ TG in erythrocyte membrane ↑ Phospholipids in erythrocyte membrane ↓ HMG-CoA: Mevalonate ratio in liver	[91]
C57BL/6J mice	Triterpene alcohol and sterol from rice bran	0.5, 2.5, 5, and 12.5 μg of cycloartenol/g BW, 23 weeks	↓ Secretion of diet-induced GIP ↓ Weight gain ↑ Fatty acid oxidation-associated gene expression, fatty acid utilization ↓ Fatty acid synthesis-associated gene expression	[92]
Male Sprague–Dawley rats	γ-Oryzanol (OZ) and ferulic acid (FA)	0.05% FA or 0.16% OZ for 13 weeks	Improved obesity, insulin resistance, and lipid profile ↓ TG, C-RP, IL-6 ↑ Adiponectin	[93]
C57BL/6J mice	Rice bran unsaponifiable matter (USM)	10 or 20, or 50 mg/kg BW/day for 6 weeks.	↓ Weight gain, food efficiency ratio, epididymal fat tissue size ↓ TG, TC, LDL-C, cardiac risk factor, and atherogenic index	[94]
Male Sprague–Dawley rats	Rice bran water extract (RBWE)	2205 mg/kg/day for 4 weeks.	↓ Body weight, visceral fat tissue weights, BGL, TC, and malondialdehyde levels ↑ Expression of eNOS ↓ Expression of NF-kB p65 and CD36	[95]
Male Sprague–Dawley rats	RBWE	2.205 or 4.410 g/kg/day for 4 weeks	↓ Expression of SREBP-1c ↑ Expression of IRS-2, GLUT-2, and GK in the pancreas ↓ Fat droplets in acinar cells ↓ HFD-induced obesity and hyperglycemia Improved glucose tolerance and TG level	[96]
Mice	Red rice bran extract (RRBE)	0.5 or 1 g/kg of RRBE for 6 weeks	↓ Adipocyte hypertrophy, lipid accumulation, and inflammation ↓ Expression of CCAAT/enhancer binding protein-alpha, sterol regulatory element-binding protein-1c, hormone-sensitive lipase, macrophage marker F4/80, NF-kB p65, monocyte chemoattractant protein-1, TNF-α, and iNOS	[97]
Male ICR mice	Red rice bran ethanolic extract (RRBEE)	0.5 or 1 g/kg BW for 12 weeks	↑ Expression of IRS and GLUT in the adipose tissue ↑ Expression of GLUT in the muscles ↓ Serum insulin level ↓ Expression of IDE in muscles ↓ Expression of pancreatic insulin and pancreatic islet size.	[98]
Mice	Rice bran (RB) or fermented rice bran (FRB)	High-fat diet with 5% of FRB or RB for 10 weeks	↓ Body weight, TG, TC, Non-HDL-C, fat cell ↑ HDL-C, adiponectin level ↓ C/EBPα, SREBP-1c, FAS, ACC	[99]
C57BL/6 male mice	Rice bran oil (RBO)	170 g of RBO/Kg of food (no changes in food consumption between groups); 10 weeks	↓ Epididymal white adipose tissue (EWAT) weights ↓ Expression of SREBP-1c and PPAR-γ in EWAT ↓ Expression of M2-macrophage markers (iNOS, COX-2, and f4/80) in EWAT ↑ Expression of arg1 and ym1 in EWAT Altered the expression of surface M2 makers (CD206 and CD11c) ↓ Expression of pro-inflammatory cytokines (IL-6 and TNF-α) ↑ Expression of anti-inflammatory cytokine (IL-10)	[100]
Male Sprague–Dawley rats	Rice bran	2 or 4 or 8% in food for 8 weeks	↓ Body weight and adipocyte size ↓ TG and TC levels in liver ↓ Glucose and uric acid in serum ↑ Phosphatidylcholine, cholesteryl ester, glycerol-1-2-hexadecanoate 3-octadecanoate levels in the liver	[101]
Male Sprague–Dawley rats	IR-64 rice bran extract	100 or 150, or 200 mg/kg BW of RBE for 6 weeks	↓ Body weight, TG, and MDA	[102]
Male ICR mice	RBWE	220 or 1100 mg/kg BW/day for 8 weeks	↓ Diastolic blood pressure ↓ Serum and liver TNF-*α* and MDA levels ↓ NF-*κ*B levels in the liver and heart ↓ Lipid accumulation in the liver ↓ Myocardial COX-2, and MMP-9 ↓ Adipose tissue mass ↓ VEGF and MMP-2 expressions in visceral fat tissue	[103,104]
Male Wistar rats	Anthocyanin-rich black rice bran extract	100 or 200 mg/kg BW/day for 8 weeks	↓ Body weight and visceral fat weight ↓ Plasma glucose, TC, and TG levels ↓ Serum creatinine and renal cortical MDA levels Attenuates kidney injury	[105]
Male Wistar rats	Rice bran	11% rice bran in the diet for 20 weeks	↓ Body weight, body fat, and adiposity index ↓ IL-6, MDA and TNF-α ↑ SOD and CAT activities in the myocardium ↓ TG, Insulin, HOMA-IR Improved the structural and functional properties of the heart	[106]
Female C57BL/6J mice	Fermented rice bran	0.239% of FRB in the diet for 8 weeks	↓ Weight gain ↓ Abundances of *Enterococcus* and *Peptostreptococcaceae* ↓ Fecal succinic acid concentration ↑ Fumaric acid in the blood ↓ Xylitol, sorbitol, uracil, glutamic acid, and malic acid levels in the blood	[107]

DAG: Diacylglycerol; RB: Rice bran; TG: Triglycerides; TC: Total cholesterol; BF: Body fat; C-RP: C-reactive protein; TNF-α: Tumor necrosis factor-alpha; iNOS: Inducible nitric oxide synthase; COX-2: Cyclooxygenase-2; VCAM-1: Vascular cell adhesion molecule-1; IL: Interleukin; Non-HDL-C: Non-high density lipid cholesterol, TL: Total lipid; HMG-CoA: 3-hydroxy-3-methylglutaryl coenzyme A; eNOS: Endothelial NOS; NADPH: Nicotinamide adenine dinucleotide phosphate; WAT: White adipose tissue; PPARγ: Peroxisome proliferator-activated receptor gamma; Emr1: Epidermal Growth Factor (EGF) module-containing mucin-like receptor-1; BW: Body weight; GIP: Glucose-dependent insulinotropic polypeptide; LDL-C: Low-density lipoprotein cholesterol; BGL: Blood glucose levels; IRS: Insulin receptor substrate; GLUT: Glucose transporter; IDE: Insulin-degrading enzyme; RBWE: Rice bran water extract; HDL-C: High-density lipoprotein cholesterol; SREBP-1c: Sterol regulatory element-binding protein-1c; IRS-2: Insulin receptor substrate-2; GLUT-2: Glucose transporter-2, GK: Glucokinase; C/EBPα: CCAAT-enhancer-binding protein α; FAS: Fatty acid synthase; ACC: Acetyl CoA carboxylase; f4/80: M1 marker; arg1: arginase 1; ym1: chitinase-like proteins; MDA: Malondialdehyde; RRB: Raw rice bran; RRBS: RRB stored for 3 months; IRRB: Infrared radiation-stabilized rice bran; IRRBS: IRRB stored for 3 months; UCP1: Uncoupling protein 1; PGC1-α: Peroxisome proliferator-activated receptor gamma coactivator 1-alpha; PRDM16: Positive regulatory domain containing 16; ALT: Alanine aminotransferase; AST: Aspartate aminotransferase; ICR: Institute of Cancer Research; MMP-9: Matrix metalloprotease-9; MMP-2: Matrix metalloprotease-2; VEGF: Vascular endothelial growth factor; CAT: Catalase; SOD: Superoxide dismutase; HOMA-IR: Homeostatic Model Assessment for Insulin Resistance.

**Table 3:** Effects of rice bran supplementation on obesity-associated biomarkers: Results of clinical studies.

Subjects	Intervention	Dose and Duration	Results	Ref.
Obese Japanese men	RB-ASG	30–50 mg/day for 12 weeks	↓ TC ↓ LDL-C ↓ Non-HDL-C ↓ LDL/HDL ratio ↓ HbA1c ↓ Abdominal circumference ↓ Subcutaneous fat area	[21]
Borderline hypercholesterolemic Chinese subjects	Refined olive oil (ROO), blended oil 1 (BO1) *, and blended oil 2 (BO2) **	30 g of ROO or BO1 or BO2 for 8 weeks	↓ TC ↓ LDL-C ↓ TG ↓ HDL-C ↓ apoB-to-apoA1 ratio ↓ Blood pressure ↓ Serum glucose ↑ Body weight	[22]
Overweight and obese adults on a calorie-restricted diet	Pigmented rice bran (PRB) or PRB with plant sterols (PRB + PS)	30 g per day for 8 weeks	↑ Body weight loss ↓ TC ↓ LDL-C ↓ Blood pressure ↓ Serum leptin ↓ F2-isoprostane	[23]
Overweight and obese adults on an energy-restriction diet	Rice bran (RB) or rice husk (RH)	70 g of RB/day or 25 of RH/day for 12 weeks	↓ Serum hs-CRP ↓ Serum IL-6	[24]

RB-ASG: Rice bran extract containing acylated steryl glucoside; TC: Total cholesterol; LDL-C: Low-density lipoprotein cholesterol; HDL-C: High-density lipoprotein cholesterol; HbA1c: Glycated hemoglobin; IL: Interleukin; Hs-CRP: High sensitivity C-reactive protein; * BO1 consists of oryzanol: sesamin: sesamolin (8000:720:300 mg/kg of oil); ** BO2 consists of oryzanol: sesamin: sesamolin (4800:300:125 mg/kg of oil); TG: Triglycerides; ApoB: Apolipoprotein B; ApoA1: Apolipoprotein A1.

**Table 4:** The influence of rice bran and rice bran derivatives on host microbiome.

Supplements	Model	Dose and Duration	Changes in Microbiome	Ref.
BBBLBAFRB	High-fat-induced obese C57BL/6J mice	0.239% fermented rice bran for 8 weeks	↓ Unclassified family *Peptostreptococcaceae* and *Enterococcus*	[107]
RB or SLFRB	High sucrose and no-fiber-fed ICR mice	20% in the diet for 2 weeks	↑ *Bacteroidetes* and *Firmicutes* ↓ *Lachnospiraceae* and *Enterorhabdus mucosicola* ↑ α-diversity of microbiota	[108]
RBO	Daidzein and RBO-supplemented mice	10% RBO for 30 days	↑ Abundance of *Lactobacillales*	[109]
RB-HBP	High-fat-diet-fed mice	100 mg/kg/day for 14 weeks	↑ α-diversity of microbiota ↑ *Bacteroidetes/Firmicutes* ratio ↑ *Bacteroides, Allobaculum, Rikenellaceae_RC9_gut_group* and *Faecalibaculum* ↓ *Alistipes, Odoribacter*, *Butyricimonas*, *Parabacteroides*, *unclassified_f_Lachnospiraceae*, *Ruminiclostridium_9*, *Romboutsia* and *norank_f_Erysipelotrichaceae*	[110]
RRB, RRBS, IRRB, IRRBS	High-fat-diet-fed C57BL/6 mice	300 mg/kg BW/day for 39 days	↑ *Bacteroidetes* and *Bacteroidetes*/*Firmicutes* ratio ↓ *Desulfovibrio * ↑ *Akkermansia* and *Lachnospiraceae*	[111]
RB-AX	In vitro fecal fermentation	100 mg of AX	↑ *Collinsella*, *Blautia* and *Bifidobacterium* ↓ *Sutterella*, *Bilophila* and *Parabacteroides*	[121]
PRBA	BALB/c mice	50, 100, and 200 mg/kg BW for 7 days	↑ *Lachnospiraceae*, *Bacteroidaceae*, *Ruminococcaceae* ↓ *Shigella*	[112]
LFRB	STZ-induced diabetic C57BL/6J mice	0.5 or 1.0 g/kg BW for 7 days	Improved the abundance of *Dubosiella* and *Lactobacillus*.	[113]
BFRB or HSRB	BALB/c mice	10% of the diet for 15 weeks	Improved the abundance of *Roseburia*, *Lachnospiraceae* and *Clostridiales*	[114]
Rice berry bran oil exerts	Male Wistar rats	100 mg/kg BW of -γ-oryzanol 5 days/week for 10 weeks	↑ *Firmicutes*/*Bacteroidetes* ratio. Improved the gut microbiota	[115]
HSRB	Healthy adult subjects	30 g/day in the diet for 4 weeks	*Bifidobacterium* and *Ruminococcus*	[116]
Rice bran	Adults with a high risk of colorectal cancer	30 g/day in the diet for 24 weeks	↑ The abundance of *Firmicutes* and *Lactobacillus* ↑ *Firmicutes*/*Bacteroidetes* ratio. ↑ Prevotella_9, *Lactobacillales*, and *Bifidobacteria*	[117]
HSRB	Malian and Nicaraguan infants	1-to-5 g/day for 6 months	↑ *α*-diversity	[118]
HSRB	Nicaraguan and Malian weaning infants	1-to-5 g/day for 6 months	Improved the gut microbiota	[119]

RB: Rice bran; AX: Arabinoxylan; SLFRB: *Saccharomyces cerevisiae* Misaki-1 and *Lactobacillus plantarum* Sanriku-SU8-mediated fermented rice bran; RBO: Rice bran oil; BBBLBAFRB: *Bacillus amyloliquefaciens* M4, *B. subtilis* M5, *Bacillus* sp. M6, *Lactobacillus casei*, *Bifidobacterium bifidum*, and *Aspergillus oryzae-*mediated fermented rice bran; RB-HBP: Rice bran-hydrolyzed bound phenolics; RRB: Raw rice bran; RRBS: RRB stored for 3 months; IRRB: Infrared radiation-stabilized rice bran; IRRBS; IRRB stored for 3 months; BW: Body weight; PRBA: Purple red rice bran anthocyanins, LFRB: *Lactobacillus fermentum* MF423-mediated fermented rice bran; STZ: Streptozotocin; BFRB: *Bifidobacterium longum*-mediated fermented rice bran; HSRB: Heat-stabilized rice bran.

## Data Availability

No new data were created or analyzed in this study. Data sharing is not applicable to this article.
